# Supplementary material for: High risk APOL1 genotypes and kidney disease among treatment naïve HIV patients at Kano, Nigeria
Source: PLoS One. 2022 Oct 13;17(10):e0275949. doi: 10.1371/journal.pone.0275949 (PMC9560498; doi:10.1371/journal.pone.0275949)
Supplement: Attachment — Submitted filename: Response to Reviewer (2).doc [file pone.0275949.s002.doc]

Department of Medicine

Bayero University Kano

22^nd^ February 2022

The Editor

PLOS One Journal

Dear Sir

**RESPONSE TO REVIEWERS FOR MANUSCRIPT PONE-D-21-10394**

I wish to resubmit our response to reviewers’ comments regarding our manuscript PONE-D-21-10394 titled ‘HIGH RISK *APOL1* GENOTYPES AND KIDNEY DISEASE AMONG TREATMENT NAÏVE HIV POSITIVE PATIENTS AT KANO, NIGERIA’. We have responded to all the issues raised by the reviewers and those raised by the editorial team as appropriate.

| Comment | Response | Page | line |
| --- | --- | --- | --- |
| **Editorial**  Please ensure that your manuscript meets PLOS ONE's style requirements, including those for file naming. The PLOS ONE style | The manuscript has been revised to follow the PLOS one’s style requirements. |  |  |
| For studies involving humans categorized by race/ethnicity, age, disease/disabilities, religion, sex/gender, sexual orientation, or other socially constructed groupings, authors should: 1) Explicitly describe their methods of categorizing human populations, 2) Define categories in as much detail as the study protocol allows, 3) Justify their choices of definitions and categories, 4) Explain whether (and if so, how) they controlled for confounding variables such as socioeconomic status, nutrition, environmental exposures, or similar factors in their analysis, and 5) Update outmoded terms and potentially stigmatizing labels to more current, acceptable terminology. Examples: “Caucasian” should be changed to “white” or “of [Western] European descent” (as appropriate); “XXX victims” should be changed to “patients with XXX. | The term ‘Caucasian’ has been changed to People of ‘European descent’  The term ‘HIV infected natives of Northern Nigeria’ has been changed to ‘HIV infected people from the Northern part of Nigeria’  The term ‘African Americans’ has been changed to ‘people of African descent’  The term ‘African ethnic population’ has been changed to ‘African Population’.  The term ‘Ashanti group’ has been changed to ‘Ashanti population’  The term ‘Yoruba group’ was changed to ‘Yoruba population’  The term Igbo speaking population has been changed to ‘Igbo population’  The phrase ‘reported among ethnic groups’ has been changed to ‘reported among population groups’ | 3  5  1  3  4,12  12  12  12  14 | 4,7,10  1  3  6,10,14  12,21  10,17  11  21 |
| Please provide the recruitment date range in your methods section. | The recruitment date has been stated | 5 | 19,20 |
| Thank you for stating in your methods "All participants gave consent to participate." Please provide additional details regarding participant consent. In the ethics statement in the Methods and online submission information, please ensure that you have specified:   - whether consent was informed  - what type of consent you obtained (for instance, written or verbal, and if verbal, how it was documented and witnessed). | Participant consent was revised with the words ‘written and informed’ added. | 6 | 8 |
| We note that you have indicated that data from this study are available upon request. PLOS only allows data to be available upon request if there are legal or ethical restrictions on sharing data publicly. For information on unacceptable data access restrictions, | We have no ethical or legal restrictions on data sharing for the study and we have uploaded minimal anonymised data set as supporting information |  |  |
| PLOS requires an ORCID iD for the corresponding author in Editorial Manager on papers submitted after December 6th, 2016. Please ensure that you have an ORCID iD and that it is validated in Editorial Manager. To do this, go to ‘Update my Information’ (in the upper left-hand corner of the main menu), and click on the Fetch/Validate link next to the ORCID field. This will take you to the ORCID site and allow you to create a new iD or authenticate a pre-existing iD in Editorial Manager. Please see the following video for instructions on linking an ORCID iD to your Editorial Manager account: | An ORCID iD for the corresponding author has been created with ID as 0000-0001-9815-2739 |  |  |
| Please include a copy of Table 4 which you refer to in your text on page 10. | Table 4 was already in the manuscript however it was mislabeled as table 3 again and this has been rectified. | 11 | 8 |
| We note you have included two tables which you refer in the text of your manuscript, however both are labelled as Table 3. Please ensure that you label each Table by a separate number in the title and also cite the relevant table number in your text; if accepted, production will need this reference to link the reader to each Table. | Table 4 was already in the manuscript however it was mislabeled as table 3 again and this has been rectified | 11 | 8 |
| Reviewer #1: |  |  |  |
| Specific Comments: I would ask the authors to include line and page numbers in all future submissions as it makes reviewing difficult without these. Consider building a hypothesis in the introduction  Table 2 was mentioned two times and did not come complete on paper when printed.  Consider describing Table legends. | Lines and page numbers have been included in the revised manuscript with tracked changes and submitted.  A Hypothesis has built in the introduction part  The numbering of tables has been revised  Legends have been added to tables 1-4 | 4  8  9  10  11 | 22  11  4  2  5 |
| **Reviewer #2**  1. The aim of the study was to “investigate the prevalence of high-risk variants and their effects on kidney disease among HIV infected patients in Northern Nigeria with hitherto limited information despite earlier reports of high population frequencies of these alleles from the Southern part of the country.” There are a number of prevalence studies from the same country region including this one from the same centre with a much larger sample size: (Apolipoprotein-1 risk variants and associated kidney phenotypes in an adult HIV cohort in Nigeria. Wudil UJ, Aliyu MH, Prigmore HL, Ingles DJ, Ahonkhai AA, Musa BM, Muhammad H, Sani MU, Nalado AM, Abdu A, Abdussalam K, Shepherd BE, Dankishiya FS, Burgner AM, Ikizler TA, Wyatt CM, Kopp JB, Kimmel PL, Winkler CA, Wester CW.Kidney Int. 2021 Apr 23:S0085-2538(21)00430-0. So, it is unclear what added value this study has provided. Rather, I think their results are conflicting with recent results from the same centre.  Leading on to the study rationale was this statement “Genetic predilection to HIVAN has been described among African-Americans but has not been widely studied among native Africans except for a few studies such as the study among the South African black population by Kasembeli et al and another study among children in the DRC.” This is not correct as there have been several other studies e.g.: | There is no conflict between the study by Wudil et al from our center which I was part of, and the present study. The populations studied were different with the former recruiting ART experienced HIV patients and the latter ART naïve patients. There are differences in the definition of CKD in the two studies with the former using albuminuria and eGFR while this study involved kidney biopsy and therefore specific kidney diseases defined by their histopathologic characteristics. The timing of the two studies was different as well.  The statement quoted “Genetic predilection to HIVAN has been described among African-Americans but has not been widely studied among native Africans except for a few studies such as the study among the South African black population by Kasembeli et al and another study among children in the DRC.” has been revised with the word ‘except’ removed. Some of the suggested references were included in the Discussion. | 4 | 20 |
| The method section is not very detailed. For instance, where were the HIV negative patients recruited from and how?, little information is provided about the methods of the kidney biopsy and how it was reported, statistical methods are not sufficiently reported, etc The methods section needs to have more details to ensure clarity. | The methods section has been reviewed source of the controls stated  The kidney biopsy procedure included | 5  6 | 19  1-3 |
| The results section is also lacking in detail: - The tables do not have a legend to describe abbreviations within each Table - Some variables do not have units (e.g. what is the unit of measurement of CD4 count?) - Why were there two Tables labelled as “Table 3”?  - What do the empty cells in the Tables mean – “no data”, “zero”, or “not reported”?  - Why are there inconsistencies with the decimals – most of the blood pressure reported have no decimals? - In reporting the histologies, this was stated in the results section: “…and the commonest histological diagnosis was FSGS seen in 20 (37.7%) patients followed by HIVAN seen in 19 (35.9%) …” Is this primary FSGS? How did you differentiate primary FSGS in HIV positive patients from HIVAN which also has an FSGS pattern on histology? | The results section has been reviewed  -Legends have been added on all the tables  Unit of measurement for the CD4 cell counts added.  Numbering of tables was reviewed  The empty cells were removed from the tables  Decimal points were added to Blood pressure measurements  The histology reports were revised  The diagnostic criteria used for HIVAN to differentiate it with FSGS were clearly stated | 8  9  10  11  8  11  9  8  8 | 11  4  2  5  11  4  3  10  21 |
| The discussion needs to probe more into the implications of your study findings, especially relative to other recent studies in the region and in Nigeria as well as implications related to kidney disease prevalence and outcomes in Nigeria | The discussion section has been revised and the suggested references were included | 12  13 | 10,12  13, 21 |
| Minor comment: I am not sure why most of the references are very old despite there being more recent citations from the region and country. | More recent references were added as suggested. | 17 | 10-17 |
| **Reviewer 3**  In the ABSTRACT,  please explain the acronyms FSGS and HIVAN | The acronyms FSGS and HIVAN were explained in the Abstract and Introduction section | 2 | 12 |
| In the INTRODUCTION: 1. In the phrase “these variants include the APOL1 G1 allele…..and the G2 allele” the verb “include” is not the most appropriate, the allele includes the variants and not vice-versa: please explain better the concept the correct nomenclature for a protein variation is p.Ser342Gly and p.Ile384Met: please correct  in the phrase “such as the study among the South African black population by Kasembeli et al and another study among children in the DRC” please explain the acronym DRC | 1. In the introduction section, the phrase ‘these variants include the APOL1 G1 allele and the G2 allele” was rewritten as ‘These alleles includes the APOL1 G1 variant’  The protein variations have been removed  .Acronym DRC has been explained as Democratic Republic of Congo | 4  4 | 3  21 |
| In the METHODS: Which is the treatment of HIV patients (both HIV positive no CKD and HIV positive CKD)? Is there any difference between the two groups? Please explain better this subject  eGFR is Estimated Glomerular Filtration Rate: please correct  It is not specified if it has been tested the Hardy-Weinberg equilibrium: please clarify | Treatment for HIV is not in the scope of this manuscript as the patients for the study were ART naïve.  Estimated Glomerular Filtration Rate (eGFR) has been corrected  The SNP data was tested for departure from the Hardy-Weinberg equilibrium and this was stated | 5  7 | 12  11,12 |
| RESULTS: In the table 1 “Characteristics of the study participants” the unit of measure of eGFR is ml/min/1.73m2 and the unit of measure of CD4 is missing, please correct. Furthermore in my opinion it would be important to have other information such as the possible presence of coinfection (hepatitis B/C), the type of ART and the HIV viral load: please indicate whether or not you have these data, and eventually why you do not have it.  In the table 2 please explain what haplotypes G0, G1GM, G1G+, G1+M, G2 mean  In the text after table 3 you state “these associations however were not significant after adjusting for age and gender”, but the association with 2 vs 1 or 0 risk alleles is already not significant (p=0.07): please explain better this concept.  Why do you adjust only for age and gender? There are other risk factors that could be included as covariates in the model, such as CD4 count, viral load, ART type and blood pressure  There are two table 3  It is not specified how you determined that G1 and G2 are on two different chromosomes, therefore in compound heterozygosity (G1/G2), and not on the same: is it based on literature data or have you tested it? Please clarify this concept | 1.Unit of measure of eGFR was corrected as ml/min/1.73m^2^ and the unit of measure of CD4 was stated (cells/mm^3^) in.  The information on ARTs was not given because the patients were ART naïve  Coinfection with hepatitis B and C were not studied as they are not part of the study objectives  The haplotypes G0, G1^GM^, G1^G+^, G1^+M^, G2 has been explained in the legend under table 2.  The sentence ‘These associations however were not significant after adjusting for age and gender’ has been removed as adjusting for the age and the gender did not add much value to the study. Most other variables suggested were not part of the data collected such as the ART since the patients were treatment naïve at the time of inclusion and the viral load measurement is not routinely done in our setting.  The numbering of the tables has been revised accordingly  I could not locate in the manuscript where it was stated ‘that G1 and G2 are on two different chromosomes | 8  9 | 11  6-8 |
| In the DISCUSSION: In the phrase “variations in the frequencies of these alleles among diverse African ethnic populations…” was should be were, please correct and add the number of reference of the cited study by Tzur et al.  Please change the sentence “the association was also strong with HIVAN…..with a less strong association in the recessive form”, a p=0.07 is not a less strong association, is not an association at all.  What does it mean the expression “from this environment” in the sentence “this study provides the first information on the high frequencies of the APOL1 genotypes….”? Please explain better. | The word ‘was’ has been replaced with ‘were’ in the phrase variations in the frequencies of these alleles among diverse African populations.  The term ‘with a less strong association in the recessive form’ was removed and replaced with the phrase ‘although the association did not reach statistical significance in the recessive form’.  The statement has been revised. The word ‘environment’ replaced with ‘this part of the country’ | 12  14  14 | 21  7-8  22 |

Your Faithfully

Aliyu Abdu
